# Sex Modifies the Association of Fibroblast Growth Factor 21 With Subclinical Carotid Atherosclerosis

**DOI:** 10.3389/fcvm.2021.627691

**Published:** 2021-04-29

**Authors:** Yingjie Chee, Grace Lx Toh, Chien Joo Lim, Liuh Ling Goh, Rinkoo Dalan

**Affiliations:** ^1^Tan Tock Seng Hospital, Singapore, Singapore; ^2^Department of Metabolic Medicine, Lee Kong Chian School of Medicine, Nanyang Technological University, Singapore, Singapore

**Keywords:** FGF-21, diabetes, carotid atheromatosis, inflammation, gender

## Abstract

**Background and Aims:** Fibroblast growth factor 21 (FGF21), an emerging metabolic hepatokine, is associated with atherosclerosis. An interaction with sex has been described in various populations. We aimed to study whether sex modulates the relationship between FGF21 and subclinical carotid atherosclerosis in a diabetes-enriched multiethnic population of Singapore. We explore differences in intermediary mechanisms, in terms of hypertension, lipids, and inflammation, between FGF21 and atherosclerosis.

**Methods:** We recruited 425 individuals from a single diabetes center in Singapore, and demographics, anthropometry, metabolic profile, FGF21, and carotid ultrasonography were performed. Multivariable logistic regression models were used to study the association between subclinical atherosclerosis and FGF21 adjusting for age, ethnicity, body mass index (BMI), hemoglobin A1c (HbA1c), systolic and diastolic blood pressures, and low-density lipoprotein (LDL)-cholesterol separately for males and females as two groups after an interaction test.

**Results:** An interaction test assessing interaction by sex on the relationship between subclinical atherosclerosis and FGF21 showed a significant interaction with sex (P_interaction_ = 0.033). In the female subgroup, significant independent associations of standardized lnFGF21 with subclinical atherosclerosis were seen, with 1 SD increment in lnFGF21 being associated with 1.48-fold (95% CI: 1.03, 2.12; *p* = 0.036) increase in risk. In the male subgroup, the association of subclinical atherosclerosis with standardized lnFGF21 was not significant [odds ratio (OR) (95% CI): 0.90 (0.63, 1.28); *p* = 0.553]. We found sex interactions with pulse pressure being significantly associated in females only and triglycerides and C-reactive protein being associated with males only.

**Conclusion:** FGF21 is positively associated with subclinical carotid atherosclerosis in women, but not in men. The sex–racial patterns in the mechanisms by which FGF21 causes subclinical atherosclerosis needs to be explored in larger population-based studies and mechanistically studied in greater detail.

## Introduction

Singapore has a population of 5.69 million (2020) with a sex ratio of ~957 males to 1,000 female residents. There are three major ethnic groups: 75% Chinese 13.7% Malays, 8.7% South Asians, and 2.6% others (http://www.singstat.gov.sg). The life expectancy in males is lower (males: 81.4 years vs. females 85.7 years). It is known that the risk of atherosclerotic diseases is generally higher in men when compared to women and higher in the Indian ethnic group compared to other ethnicities. However, emerging evidence has revealed sex disparities in the pathogenesis of coronary artery disease. In women, microvascular changes associated with systemic hypertension could confer a greater risk of developing atherosclerosis (1), whereas inflammation remains the predominant driver in men. Evaluating the impact of sex on the novel biomarkers will be crucial in personalizing cardiovascular risk assessment.

Fibroblast growth factor 21 (FGF21), an emerging metabolic hepatokine, has been shown to exert beneficial effects on critical atherosclerotic pathways (2) and has been reported to be an independent predictor of subclinical atherosclerosis (3). Beyond its well-described role in lipid and glucose metabolism, FGF21 has also demonstrated a beneficial effect on blood pressure regulation *via* the renin–angiotensin–aldosterone system. FGF21 analogs are therefore potential therapeutic targets for atherosclerosis (4). Furthermore, it is being increasingly recognized that individual response to FGF21 is highly heterogeneous. There is a need to identify genetic, phenotypic, and metabolic factors that confer FGF21 responsiveness (5).

One of these important factors is sex. Previous studies in the Singapore population show that sex modulates the association between FGF21 and new-onset diabetes and diabetic nephropathy with a positive association seen only in women (6, 7). Current studies, looking at sex differences between the association of FGF21 and carotid atherosclerosis, showed conflicting results with two studies conducted in Hongkong and China showing positive association only in women (8, 9), whereas others in Chinese and in ethnically diverse populations showing no significant sex differences (3, 10, 11).

In this cross-sectional study of a diabetes-enriched multiethnic cohort in Singapore consisting of Chinese, Malays, and Indians, we assessed whether sex modulates the relationship between FGF21 and subclinical carotid atherosclerosis and explored interactions with other traditional cardiovascular risk factors, with the aim of enhancing our understanding of FGF21 in the atherosclerotic pathways.

## Methods

In this cross-sectional study, we recruited individuals from a single tertiary hospital in Singapore. Inclusion criteria included age 21–80 years, current non-smokers. The exclusion criteria were inability to give informed consent, pregnancy, hospitalization for any condition or recent infections (last 2 weeks), estimated glomerular filtration rate (eGFR) <20 ml/min/1.73 m^2^, concomitant immunosuppressive agents or corticosteroids, malignancies, or rheumatological conditions. Fasting morning blood was collected from each participant, and this was processed and stored as plasma and serum at −80°C. Demographics, anthropometry, and metabolic profile were done as per standard methods. Serum FGF21 was measured in the stored serum samples using enzyme-linked immunoassay (Abcam) with a sensitivity of 3.3 pg/ml, intra-assay coefficient of variation 5.3%, and inter-assay coefficient of variation 5.5% in duplicates. Carotid ultrasonography was performed using a multifrequency high-resolution linear transducer probe (GE Logiq P5) by two trained operators according to the Mannheim carotid artery intima-media thickness (CIMT) consensus (12). These images were independently interpreted by two trained study investigators to identify atherosclerotic plaques. Subclinical atherosclerosis, the primary outcome, was defined as maximum average CIMT <0.8 mm or presence of plaque or both (13). This study was conducted in accordance to the Statement of Helsinki, and written consent was obtained from all participants. Ethics approval was obtained from the local institutional review board (DSRB Ref: 2007/01131).

### Statistical Analysis

Our primary objective was to examine potential associations between FGF21 and subclinical atherosclerosis. Assuming a power of 90%, a level of significance of 0.05, two-sided tests, and the mean FGF21 of 200 vs. 260 pg/ml in subclinical atherosclerosis with SD of 100 vs. 180, we would require 188 patients (94 with subclinical atherosclerosis vs. 94 with no subclinical atherosclerosis) to see a difference in each group. The patients recruited in this study were from a tertiary center, and most of the individuals recruited had an anticipated higher risk of subclinical atherosclerosis. Hence, we planned to sample at a minimum 200 males and females to study the associations in subgroups.

**Figure 1:**
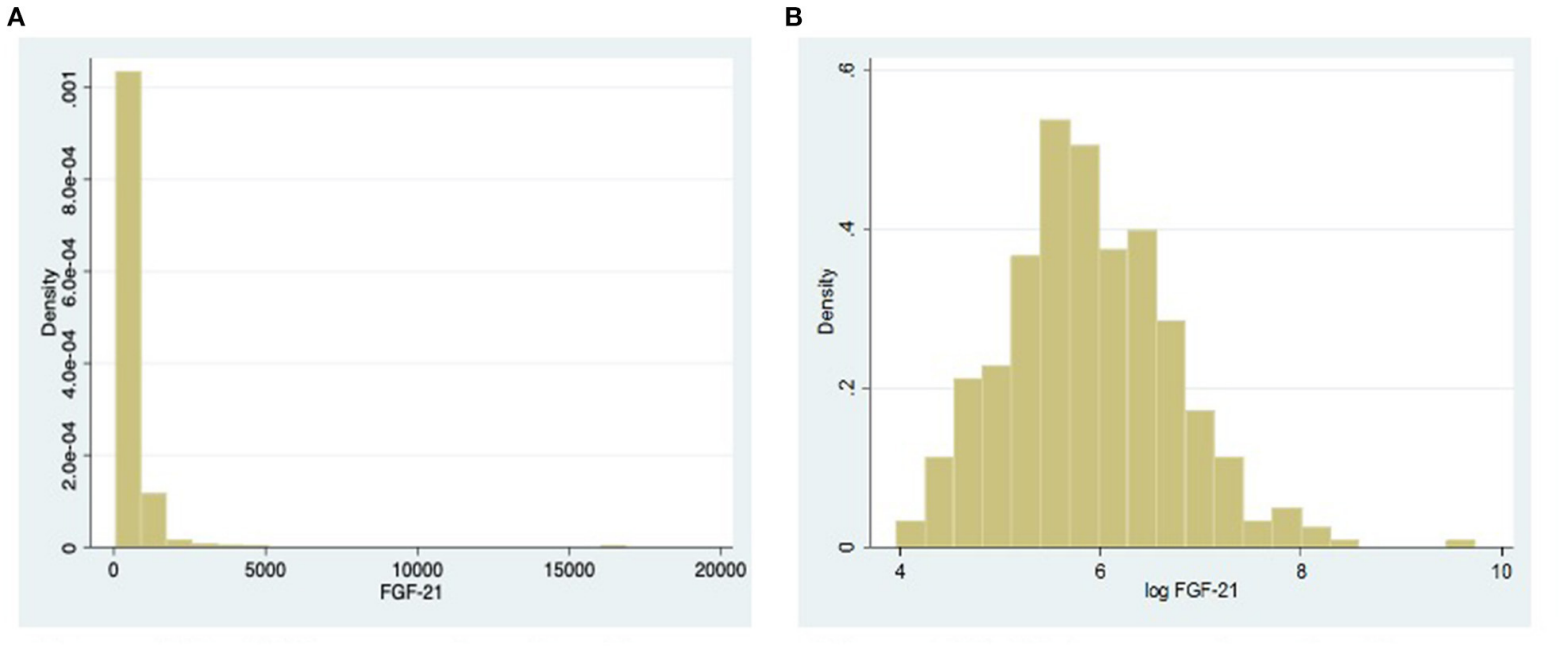
**(A)** Histogram showing the distribution of fibroblast growth factor 21 (FGF21). **(B)** Histogram showing the distribution of FGF21 after natural log transformation.

All categorical variables are described as number (percentage) and continuous variables as mean (standard deviation) for normally distributed variables and as median [interquartile range (IQR)] for skewed variables. We tested FGF21 that is the main determinant of interest in this study for distribution using the skewness and kurtosis test. Since FGF21 was very significantly skewed (*P* < 0.0001) (see histogram in Figures 1A,B), we performed natural log (ln) transformation before analysis. The interaction with sex for the relationship between subclinical atherosclerosis and lnFGF21 was tested using an interaction test (Wald's test). Multivariable logistic regression model was planned *a priori* to study the association between subclinical atherosclerosis and FGF21, adjusting for age, ethnicity, body mass index (BMI), hemoglobin A1c (HbA1c), systolic and diastolic blood pressures, and low-density lipoprotein (LDL)-cholesterol separately for males and females as two groups. *Post hoc*, we analyzed the interaction effects of sex in the relationship between blood pressure, lipids, and inflammation with FGF21 to explore possible intermediary mechanisms in the two groups using linear regression. All statistical analyses were done using Stata version 16.

## Results

We recruited 203 (47.8%) males and 222 (52.2%) females. The mean age of females was 51 (13) years. Approximately 62% (137) of the women were ≥49 years of age and postmenopausal. Among the 425 individuals, 375 individuals were known to have type 2 diabetes mellitus and 111 (26%) belonged to Indian ethnicity. All cardiometabolic variables such as age, BMI, systolic and diastolic blood pressures, HbA1c, and lipid profiles were similar in males and females. In the female subgroup, median (IQR) FGF21 concentrations, 355.7 (223.1–671.2) pg/ml, and C-reactive protein (CRP) concentrations, 2.2 (0.9, 4.2), were higher than those in the male subgroup: FGF21: 326.2 (190.5–584.6) pg/ml; CRP: 1.1 (0.5, 2.9). The other baseline characteristics are described in Table 1. Subclinical atherosclerosis was present in 110 (54.2%) males and 90 (40.5%) females.

**Table 1:** Baseline characteristics of the participants.

**Total *n* = 425**	**All**	**Males**	**Females**	**Males**		**Females**	
	***N* = 425**	***N* = 203 (47.8)**	***N* = 222 (52.2)**	**No SA = 93 (45.8)**	**SA = 110 (54.2)**	**No SA = 132 (59.5)**	**SA = 90 (40.5)**
Age, Mean (SD)	51.7 (12.8)	52.4 (12.2)	51.1 (13.4)	48.0 (13.3)	56.1 (9.9)	46.5 (14.2)	57.9 (8.4)
Ethnicity, n (%)							
Chinese	256 (60.2)	139 (68.5)	117 (52.7)	65 (69.9)	74 (67.3)	71 (53.8)	46 (51.1)
Malays	58 (13.7)	22 (10.8)	36 (16.2)	13 (14.0)	9 (8.2)	28 (21.2)	8 (8.9)
Indians	111 (26)	42 (20.7)	69 (31.1)	15 (16.1)	27 (24.5)	33 (25.0)	36 (40.0)
DM, n (%)	375 (88)	186 (92)	189 (85)	79 (85)	107 (97)	101 (76.5)	88 (97.7)
BMI, kg/cm^2^, Mean (SD)	30.0 (5.2)	27.0 (5.1)	26.9 (5.3)	27.2 (6.0)	26.9 (4.2)	26.5 (5.7)	27.5 (4.5)
Systolic BP, mm Hg, Mean (SD)	131.4 (17.2)	131.8 (15.6)	131.0 (18.6)	129.6 (15.7)	133.6 (15.3)	126.5 (16.3)	137.65 (19.9)
Diastolic BP, mm Hg, Mean (SD)	74.3 (9.1)	76.5 (8.9)	72.3 (8.9)	76.4 (10.1)	76.5 (7.7)	72.2 (8.9)	72.5 (8.9)
HbA1c %, mean (SD)	8.2 (2.0)	8.3 (1.8)	8.2 (2.1)	8.0 (1.9)	8.6 (1.8)	7.9 (2.1)	8.6 (1.9)
Total Cholesterol, mmol/L, Mean (SD)	4.4 (1.0)	4.3 (1.0)	4.6 (1.0)	4.2 (0.9)	4.3 (1.0)	4.6 (1.1)	4.6 (0.9)
HDL-C, mmol/L Mean (SD)	1.2 (0.3)	1.1 (0.2)	1.3 (0.3)	4.2 (0.9)	1.0 (0.2)	1.3 (0.4)	1.3 (0.3)
LDL-C, mmol/L Mean (SD)	2.6 (0.8)	2.5 (0.8)	2.6 (0.8)	2.4 (0.8)	2.6 (0.9)	2.7 (0.8)	2.6 (0.8)
Triglyceride, mmol/L Mean (SD)	1.7 (1.9)	1.8 (1.6)	1.6 (2.2)	1.8 (1.9)	1.7 (1.2)	1.7 (2.7)	1.5 (0.9)
CRP, mg/L Median (IQR)	1.5 (0.63, 7.0)	1.1 (0.5, 2.9)	2.2 (0.9, 4.2)	1.1 (0.5, 2.4)	1.15 (0.5, 3.0)	1.9 (0.7, 3.8)	2.6 (1.1, 4.9)
CRP, mg/L Mean (SD)	3.0 (3.9)	2.2 (2.7)	3.6 (4.6)	2.2 (2.9)	2.2 (2.5)	3.5 (4.8)	3.9 (4.3)
FGF-21, pg/ml Median (IQR)	348.0 (210.6, 616.4)	326.2 (190.5, 584.6)	355.7 (223.1–671.2)	326.2 (151.1, 611.9)	322.4 (202.6, 536.9)	311.5 (215.6, 584.4)	492.3 (266.43, 769.6)
FGF-21, pg/ml Mean (SD)	542.9 (958.7)	485.4 (575.2)	595.4 (1206.2)	559.8 (766)	422.6 (330.6)	474.6 (502.9)	772.4 (1785.4)

*SA, subclinical atherosclerosis; SD, standard deviation; IQR, interquartile range; BP, blood pressure; DM, diabetes mellitus; BMI, body mass index; CRP, C-reactive protein; FGF-21, fibroblast growth factor-21*.

An interaction test assessing interaction by sex on the relationship between subclinical atherosclerosis and FGF21 showed a significant interaction with sex (P_interaction_ = 0.033).

**Figure 2:**
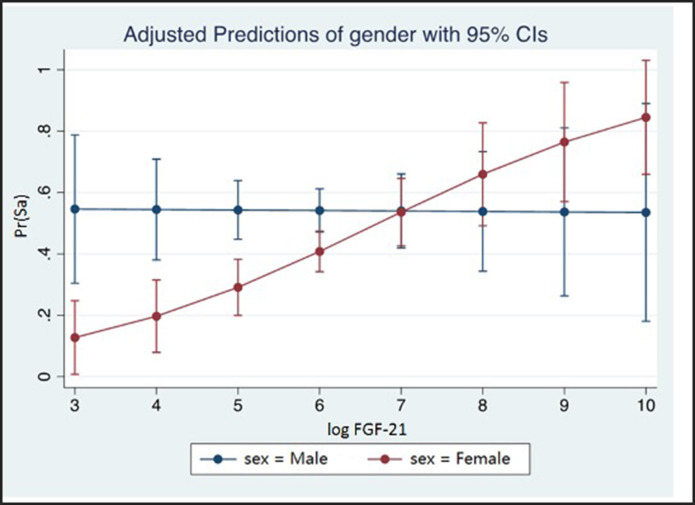
The probability of subclinical atherosclerosis with 95% CI shown at different levels of standardized lnFGF21 in males and females. lnFGF21, standardized natural log fibroblast growth factor 21; SA, subclinical atherosclerosis; interaction test *p*-value = 0.033.

Multivariable logistic regression analysis in the female subgroup showed significant independent associations of standardized lnFGF21 with subclinical atherosclerosis, with 1 SD increment in lnFGF21 being associated with 1.48-fold (95% CI: 1.03, 2.12; *p* = 0.036) increase in risk. In the male subgroup, the association of subclinical atherosclerosis with standardized lnFGF21 was not significant [adjusted odds ratio (OR) (95%CI): 0.90 (0.63, 1.28); p = 0.553) (Table 2, Figure 2)].

**Table 2:** Multivariable logistic regression for subclinical atherosclerosis with standardized lnFGF21 adjusted for age, ethnicity, BMI, blood pressure, LDL-cholesterol, and glycated hemoglobin.

**Total *n* = 425**	**Males**, ***n*** **=** **203**		**Females**, ***n*** **=** **222**	
	**Adj. Odds Ratio (95% CI)**	***P*-Value**	**Adj. Odds Ratio (95% CI)**	***P*-Value**
lnFGF21^∧^	0.90 (0.63, 1.28)	0.553	1.48 (1.03, 2.12)	0.036*
Age (year)	1.07 (1.04, 1.11)	<0.001*	1.07 (1.03, 1.11)	<0.001*
Ethnicity :				
Chinese	Ref		Ref	
Malays	0.47 (0.15, 1.48)	0.196	0.46 (0.17, 1.30)	0.143
Indians	1.46 (0.65, 3.27)	0.361	0.82 (0.39, 1.74)	0.608
BMI, kg/cm^2^	1.04 (0.97, 1.12)	0.276	1.01 (0.95, 1.09)	0.699
Systolic BP, mm Hg	1.01 (0.99, 1.04)	0.342	1.03 (1.00, 1.06)	0.028*
Diastolic BP, mm HG	1.00 (0.95, 1.05)	0.892	0.97 (0.92, 1.02)	0.202
LDL-Cholesterol, mmol/L	1.88 (1.21, 2.90)	0.005*	1.55 (1.02, 2.37)	0.041*
HbA1c, %	1.08 (0.89, 1.31)	0.448	1.03 (0.87, 1.22)	0.724

∧ lnFGF21, standardized natural log fibroblast growth factor 21; BMI, body mass index; BP, blood pressure; LDL, low-density lipoprotein; HbA1c, glycated hemoglobin; *p value <0.05.

### *Post hoc* Exploratory Analysis

**Figure 3:**
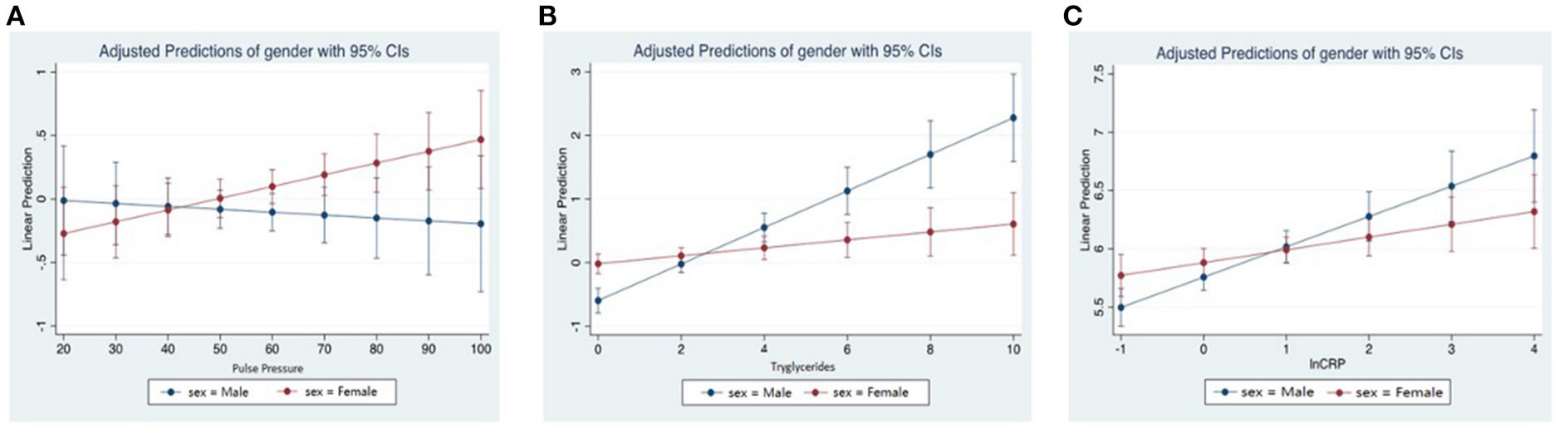
**(A)** Linear predictions of standardized natural log fibroblast growth factor 21 (lnFGF21) with pulse pressure by sex. **(B)** Linear predictions of lnFGF21 with triglycerides by sex. **(C)** Linear predictions of lnFGF21 with lnCRP by sex.

We assessed the relationship of lnFGF21 with blood pressure in female and male subgroups. We found a significant linear association of lnFGF21 with systolic blood pressure in females only [females: 0.006 (0.001, 0.012); *p* = 0.029 vs. males: 0.004 (−0.003, 0.011)] and diastolic blood pressure in males only [females: 0.007 (−0.005, 0.019); *p* = 0.227 vs. males: 0.016 (0.003, 0.029); *p* = 0.015]. When we assessed pulse pressure in the different sex subgroups, we found that lnFGF21 was positively linearly associated with pulse pressure in females only [Coeff: 2.52 (95% CI: 0.14, 4.90); *p* = 0.038], whereas in the males, this relationship was not seen [Coeff: −0.39 (95% CI: −2.38, 1.60); *p* = 0.700] (Figure 3A).

An interaction effect with sex was seen in the lnFGF21 with triglycerides (P_interaction_ < 0.001), and a significantly higher association was seen in the males [Coeff (95% CI): 0.24 (0.17–0.30); *p* < 0.001] when compared to females [0.05 (0.002, 0.10); *p* = 0.041] (Figure 3B).

An interaction test assessing interaction by sex on the relationship between CRP and lnFGF21 showed a significant interaction with sex (P_interaction_ = 0.027). In subgroup models, correlations between lnCRP and lnFGF21 in the males [Coeff: 0.26 (95% CI: 0.16, 0.36; *p* < 0.001] were stronger than those in the females [Coeff: 0.11 (95% CI: 0.02, 0.20); *p* = 0.018] (Figure 3C).

No sex interaction effects were seen with age, BMI, and HbA1c.

## Discussion

In this study, we provide further evidence that FGF21 is positively associated with subclinical atherosclerosis in women, but not in men, in a diabetes-enriched multiethnic cohort in Singapore. These sex differences are similar to those of other population-based studies conducted in Singapore in terms of nephropathy (7) and in terms of diabetes incidence (6) and in China in terms of subclinical atherosclerosis and lower extremity atherosclerotic disease in diabetes patients (9, 14). Other studies in Chinese and in ethnically diverse populations have shown no significant sex differences (3, 10, 11). In a recent study from China with a prospective follow-up, composed of a mixed group of individuals, although a correlation with subclinical atherosclerosis independent of fatty liver status was described, sex interactions were not seen (3). Overall, more studies that included type 2 diabetes participants (4, 5, 7, 12) showed significant sex interactions, whereas studies involving healthy population groups showed no correlations.

The biological mechanisms underpinning this sex dimorphism remains elusive. It could be related to estrogen status, as it has been seen that activation of hepatic estrogen receptor-α increases energy expenditure by stimulating the production of FGF21 in female mice (15). In one study, baseline FGF21 levels were higher in females, and the levels decreased from baseline levels after increased fructose consumption in women only, suggestive of a possible difference in both secretion and sensitivity between the sex groups (16). A higher concentration of FGF21 has also been reported in female Danish children and adolescents, which was attributed to higher concentrations of triglycerides in females (17).

At the molecular level, FGF21 has shown protective effects on blood pressure regulation by inducing angiotensin-converting enzyme (ACE) 2 and its downstream angiotensinogen (1–7) production (4). In animal models, overexpression of FGF21 was observed in hypertensive mice with consequent blood pressure lowering and improved vascular function. There could be a sex disparity in the beneficial effect of FGF21 on blood pressure regulation. Female mice displayed a significantly higher expression of FGF21 and FGF21 receptors and achieved more potent BP reduction in response to recombinant FGF21 administration compared to males (18). At the population level, FGF21 has been independently associated with hypertension (19), although a distinction between sex has not been reported. In our study, subgroup analysis revealed a significant relationship of systolic blood pressure and pulse pressure in females only. However, despite adjustment for systolic blood pressure and pulse pressure, FGF21 remained independently associated with subclinical atherosclerosis in females, thus suggesting other possible interacting factors as well. Moreover, brachial-ankle pulse-wave velocity, a measure of arterial stiffness, has been correlated with serum FGF21 concentrations in women, and the concentrations have been seen to decrease with improvement of brachial-ankle pulse-wave velocity measurements (20). FGF21 was also associated with femoral intima-media thickness and peripheral arterial disease in Chinese women but not in men (14). Since FGF21 plays a crucial role in blood pressure regulation *via* the renin–angiotensin–aldosterone system, it is possible that the long-term higher expression of FGF21 and potential for higher modulation of blood pressure and arterial stiffness in females contribute to the stronger relationship between FGF21 and may influence vascular function, intima-media thickening, and remodeling as compared to males in the pathogenesis of atherosclerosis.

In terms of inflammation, we did see a stronger significant association of FGF21 with inflammation in males. Addition of CRP as a covariate in the model also did not change the results in the female subgroup, showing that FGF21 associated with carotid atherosclerosis independent of CRP concentrations or inflammation. In a large-scale community screening for peripheral artery disease (PAD), the higher prevalence of PAD among women could not be accounted for despite having higher CRP. On the contrary, a strong association between CRP and PAD was observed in men (21). A genome-wide analysis of carotid plaque burden revealed a significant hit for men at 5q31.1 (rs201629990), which points toward a role of interleukin-5 (IL5), an inflammation marker, in men only (22). Several estrogen response elements in this locus point toward a functional explanation of the observed sex-specific effect (22). Our findings thus suggest that the sex dichotomy in atherosclerosis pathogenesis could be mediated by vastly different mechanisms. The association between FGF21 with subclinical atherosclerosis in women independent of markers of inflammation may suggest other pathways, for example, involving vascular hemodynamics, as the dominant process in women. This may in turn fine-tune the role of FGF21 as a biomarker of vascular function in women and may translate to different therapeutic purposes of FGF21 analogs between sexes.

Our study has several limitations. This is a cross-sectional study that would limit determination of a causal relationship between FGF21, sex, and subclinical atherosclerosis. Since the study was not designed to study the effects of ethnicity, we were not able to study ethnicity-specific patterns in detail. We did not measure estrogen concentrations, and associations with the estrogen status cannot be determined. We did not have follow-up data for hard macrovascular outcomes; hence, it is not possible to determine associations with outcomes. We did not adjust for multiple hypotheses testing. The relatively small number of individuals recruited from a single tertiary center may limit the generalizability of the results.

## Conclusion

**Figure 4:**
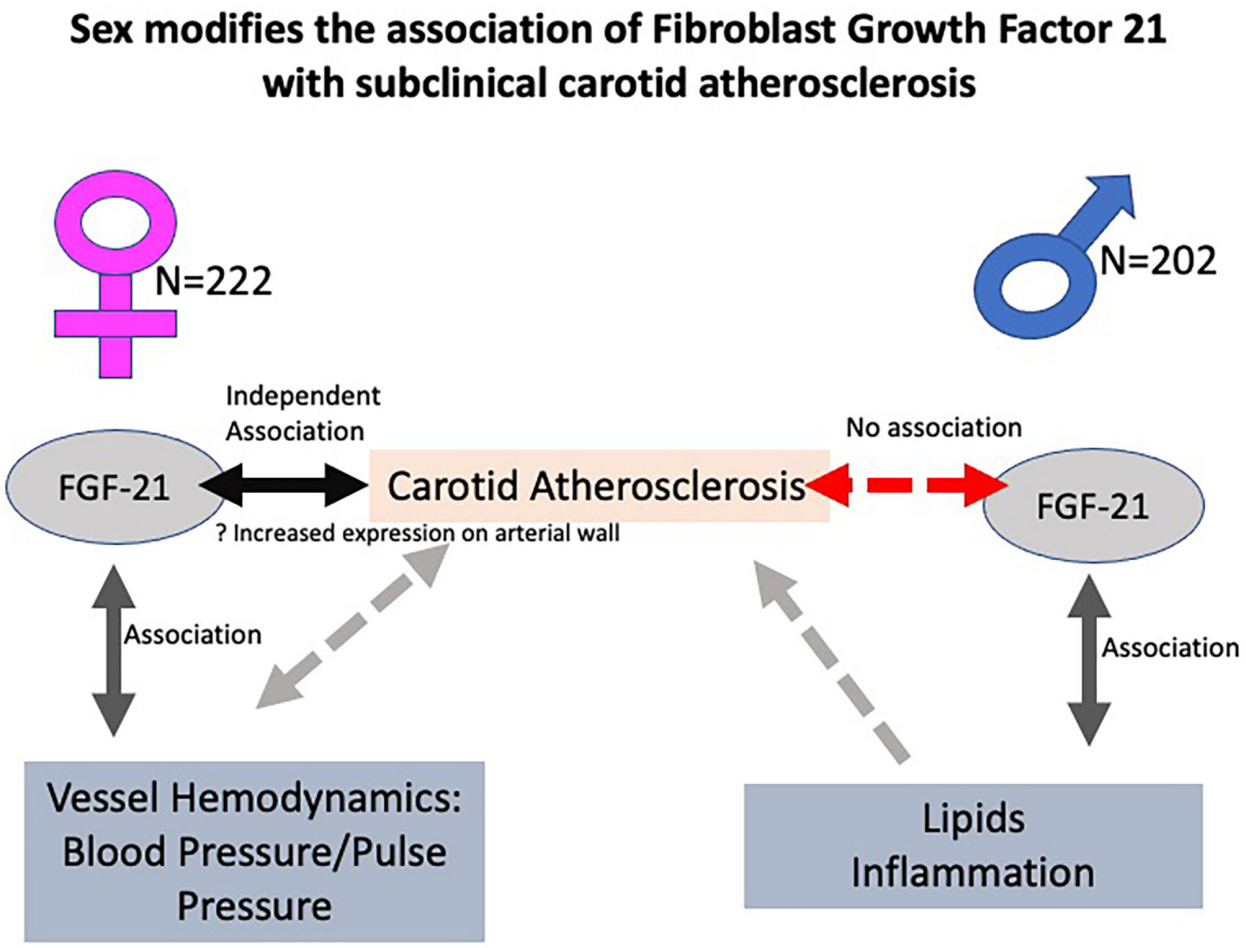
Central illustration showing the sex interactions in the relationship between fibroblast growth factor 21 (FGF21) and carotid atherosclerosis with postulated mechanisms. FGF21 is positively associated with subclinical carotid atherosclerosis in women but not in men. A postulated hypothesis is that a downstream signaling of FGF21 on vascular hemodynamics like blood pressure and vascular stiffness is likely responsible for this effect in women. In men, likely FGF21 affects inflammation and lipids, which is known to cause atherosclerosis.

FGF21 is positively associated with subclinical carotid atherosclerosis in women but not in men. Our exploratory analysis suggests a hypothesis that a downstream signaling of FGF21 on vascular hemodynamics like blood pressure and vascular stiffness is likely responsible for this effect in women. In men, likely FGF21 affects inflammation and lipids, which is known to play a significant role toward the development of carotid atherosclerosis (Figure 4). This sex–racial pattern in tissue specificity in FGF21 sensitivity and tissue specificity needs to be explored in greater detail in larger population-based studies. Further understanding of these mechanisms would be crucial in the planning of personalized therapeutics.

## Data Availability Statement

The raw data supporting the conclusions of this article will be made available by the authors, without undue reservation.

## Ethics Statement

The studies involving human participants were reviewed and approved by Domain Specific Review Board, National Healthcare Group. The patients/participants provided their written informed consent to participate in this study.

## Author Contributions

YC conceived the idea, conducted the study and wrote the first draft of the manuscript. GT and LG performed the ELISA measurements of FGF-21, reviewed and critically evaluated the manuscript. RD conceived the idea, conducted the study, analyzed the data and wrote the manuscript. All authors reviewed the final manuscript.

## Conflict of Interest

The authors declare that the research was conducted in the absence of any commercial or financial relationships that could be construed as a potential conflict of interest.
